# Statistical and Graphical Assessment of Circumferential and Radial Hardness Variation of AISI 4140, AISI 1020 and AA 6082 Aluminum Alloy

**DOI:** 10.3390/ma5010012

**Published:** 2011-12-23

**Authors:** Hamad Al-Khalid, Ayman Alaskari, Samy Oraby

**Affiliations:** Department of Mechanical Production Technology, College of Technological Studies, The Public Authority of Applied Education and Training (PAAET), P.O. Box 38322, Dhaya Abdalla Alsalim 72254, Kuwait; E-Mails: hk.alkhalid@paaet.edu.kw (H.A.-K.); aalaskai@gmail.com (A.A.)

**Keywords:** hardness variation, AISI 4140, AISI 1020, AA 6082, reference block calibration, statistical analysis, response surface

## Abstract

Hardness homogeneity of the commonly used structural ferrous and nonferrous engineering materials is of vital importance in the design stage, therefore, reliable information regarding material properties homogeneity should be validated and any deviation should be addressed. In the current study the hardness variation, over wide spectrum radial locations of some ferrous and nonferrous structural engineering materials, was investigated. Measurements were performed over both faces (cross-section) of each stock bar according to a pre-specified stratified design, ensuring the coverage of the entire area both in radial and circumferential directions. Additionally the credibility of the apparatus and measuring procedures were examined through a statistically based calibration process of the hardness reference block. Statistical and response surface graphical analysis are used to examine the nature, adequacy and significance of the measured hardness values. Calibration of the apparatus reference block proved the reliability of the measuring system, where no strong evidence was found against the stochastic nature of hardness measures over the various stratified locations. Also, outlier elimination procedures were proved to be beneficial only at fewer measured points. Hardness measurements showed a dispersion domain that is within the acceptable confidence interval. For AISI 4140 and AISI 1020 steels, hardness is found to have a slight decrease trend as the diameter is reduced, while an opposite behavior is observed for AA 6082 aluminum alloy. However, no definite significant behavior was noticed regarding the effect of the sector sequence (circumferential direction).

## 1. Introduction

In all industries, it is desired to introduce a better product quality to the market. The quality of the product usually represents the processing effectiveness of three stages: materials selection, design and manufacturing processes. At all those stages, the uniformity of the raw material properties is assumed to be provided by the manufacturer. Moreover, the properties of the raw material are not necessarily homogenous after processing or manufacturing of the product [1,2,3]. There are different reasons for deviation and nonuniformity of the raw materials provided by the manufacturer. A soluble unequal proportion of chemical elements in the alloy is one of the most important reasons for the different properties of the raw material. Improper heat treatment especially for thicker material leads to different microstructure phases across the workpiece material each has its own properties. Also surface finishing and preparation changes the surface properties of the workpiece [4,5,6].

Knowledge of material properties should be available to consider the effects of undergoing loading conditions not only on the product outer surface but also in the zone on specified distance from the surface. Among the important material properties, hardness represents one of the most important factors affecting product performance and its durability. Hardness data provide the basis for determining the hardening coefficient, the residual stress, the degree of surface layer destruction, the yield point, the strength, and the true fracture strength of the material.

In this paper, the hardness variations over the different diameters of some ferrous and nonferrous structural engineering materials; such as-hot-rolled AISI 1020 and AISI 4140 quenched and tempered hot alloy steel and AA6082 aluminum alloy in T6 conditions, were investigated. Measurements were performed over both faces (cross-section) of each stock bar according to a prespecified stratified design ensuring the coverage of the entire area both in diagonal and circumferential directions. Additionally, the credibility of the apparatus and measuring procedures were examined through a statistically based calibration process of the hardness reference block.

Statistical criteria, such as Descriptive, *T*-test Pairs, One-Way ANOVA and partial correlation measures were used to detect the possible dependency of hardness values with the testing location over the specified tested surface either diagonally (radial) or circumferentially. Graphical response surface, surface map and contours were used to describe the data and to detect any possible trends.

## 2. Experimental Setup and Instrumentation

For universality purposes, three different types of materials were used in this study in the form of a 200 mm length and 150 mm diameter round bars. Specifications, mechanical properties and chemical composition, as provided by manufacturer, are listed in Table 1. Both sides (faces) of each stock bar were finished by low-feed-high speed face turning to create a flat surface with acceptable quality. Further surface preparation was carried out using a very fine sandpaper so that the minimum finishing requirement (Ra ≤ 1mm) is maintained where the measured Ra range over both surface was found to be (0.97–1.04).

**Table 1:** Mechanical properties and chemical composition of the employed materials.

Material	Mechanical physical properties				Chemical composition (wt %)										
	Tensile Stren. (MPa)	Yield Stren. (MPa)	Hardn. (HRB)	Elo. (%)	C	Mn	Si	P	S	Cr	Mo	Ni			
AISI 1020	485		79		0.22	0.47	0.17	0.012	0.004						
AISI 4140	1093	945	99	14	0.41	0.88	0.28	0.018	0.009	1.01	0.18	0.10			
	**U.T.S**	**0.2 P.S.**			**Si**	**Fe**	**Ti**	**Mn**	**Mg**	**Cu**	**Zn**	**Zr**	**Cr**	**Ni**	**Va**
AA6082	326.6	273		8	1.04	0.19	0.018	0.58	0.8	0.025	0.003	0.001	0.003	0.001	0.004

Stationary hardness testers can only accommodate test pieces of limited size. Moreover, transportation of the test pieces is often impractical, sometime impossible therefore; the portable hardness tester can give a key solution. In the current study, hardness testing was carried out using a COMPUTEST SC digital Rockwell portable Tester with many advanced features [7]. Instrument and measuring features conform to both ASTM B724 and DIN 50157 standards. Measuring range and scales include HRC (0–70) and HRB (0–120) using a static [5 kg (49N)] load and with preload of [1.2 kg (11.8 N)] according to the Rockwell principle. Features are found appropriate for measuring the HRB nominal hardness values of the three tested materials. Device calibration and measurement credibility were evaluated in details using its reference block.

## 3. Results, Analysis and Discussion

In order to obtain significant outcome, indentation locations over the intended surface should be carefully determined through the proper stratified design with the consideration of the trend and frequency of hardness distribution in both the radial and circumferential directions. Data evaluation included both of the descriptive graphical response surface methodology, to visualize the individual and the interaction of the involved parameters and the appropriate statistical parameters such as Means, *T*-Test, one-way ANOVA and Correlation, to determine the adequacy and significance of possible evolving trends.

### 3.1. Apparatus Calibration and Hardness Variability of the Reference Block

**Figure 1:**
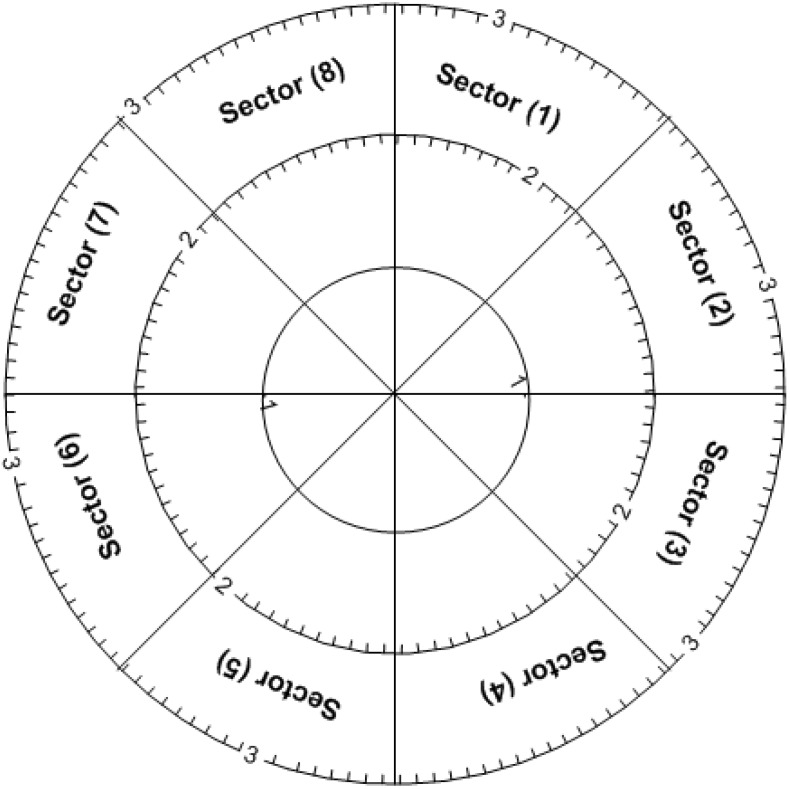
Stratifying design for hardness reference block.

Calibration of the measuring device using its reference block is one of the important factors affecting the hardness measurement. The hardness reference block is of ultimate importance, supplying the criteria to determine the standard value of a product, to verify hardness of testing machines and to specify accuracy of quality control measurements. This procedure may provide the mutual benefit of examining the accuracy of both the reference block and the measuring device. Therefore, to increase the credibility of the recorded hardness, the device was calibrated using the reference hardness block that is provided by manufacturer. As shown in Figure 1, the indentation locations were determined as the intersection of a stratified design that includes eight circumferential sectors and three radial layers. This led to 24 indentation locations to give a high level of reliability and repeatability of the measuring device. According to ISO 6507—part 3 [1,2,3], only five indentations are required as a minimum number. Many other investigations [6,8,9] have been carried out to determine the best way of the reference block calibration and its uncertainty. It has been reported [9] that more than 6 or even 12 strata were recommended for reliable hardness measurement since, in their opinion, only five indentations proposed by [8] did not seem to be sufficient to gain statistically reliable calibration values.

In order to find out how to locate the test indentation positions and, at the same time, to determine the number of indentations sufficient for a significant measure, the reference block was mapped as indicated in Figure 1. The surface of the reference block was divided into eight circumferential sectors and three diagonal concentric circles (layers). This yielded a stratification design of a total of 24 hardness indentations map locations. The influence of stratified conditions on the measured hardness values was statistically and graphically judged.

General descriptive statistics of the measurement is listed in Table 2. Although the data mean value (81.74 HRB) is very close to the nominal value (82 HRB), some experimental error and outliers have affected the results and their statistical features. However, the “Detect Anomaly” features in the SPSS statistical program is used to identify the unusual cases within the data. The Anomaly Detection procedure searches for unusual cases based on deviations from the norms of their cluster groups. The algorithm is designed for generic anomaly detection; that is, the definition of an anomalous case is not specific to any particular application. Algorithm steps are usually: Modeling, Scoring and Reasoning. More details about the procedures can be found in [10].

**Table 2:** Descriptive statistics of the reference block hardness measurement.

	No. Cases		Minimum		Maximum		Mean		Std. Dev.	
	Entire data	Filtered data	Entire data	Filtered data	Entire data	Filtered data	Entire data	Filtered data	Entire data	Filtered data
H_i_	24	16	67.90	76.3	96.10	87.00	81.74	82.90	6.644	3.090

**Figure 2:**
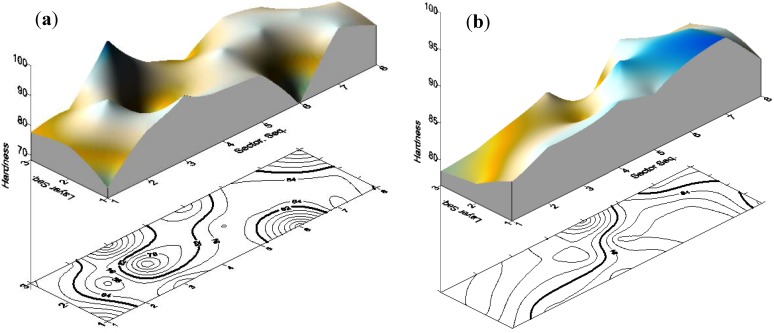
Response surface of the hardness measurement of the reference block. (**a**) Unfiltered data; and (**b**) Filtered data.

**Figure 3:**
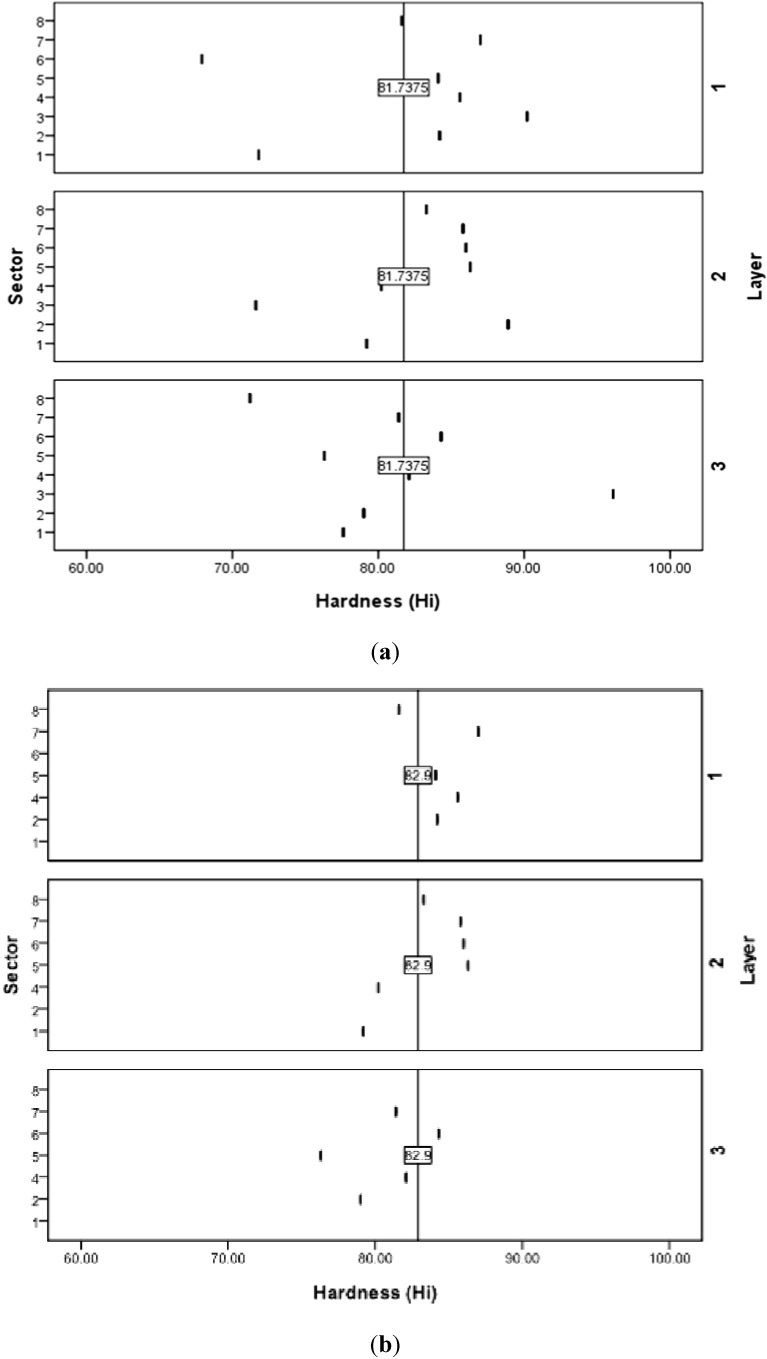
Hardness map distribution over sector and layer. (**a**) Unfiltered data; and (**b**) Filtered data.

A criterion 5% exclusion percentage with the highest anomaly index value is selected for the outlier identification. Tracking out and removal of the eight detected outlier data points has enormously improved the data trends and their statistical measures. Considering that i and j are the sector and layer sequence respectively, the removed eight outlier points p(i,j) were: (1,1), (1,3), (2,2), (3,1), (3,2), (3,3), (6,1), (8,3). As listed in Table 2, the standard deviation was reduced from 6.644 for entire data (unfiltered) to only 3.09 for the filtered data. Data enhancement can be further observed through the data distribution over the sector-layer response surface and contour plots as shown in Figure 2. For the unfiltered data, Figure 2(a), higher measured hardness values can be observed especially at sector numbers 3 and 6. Outliers removal led to less variation and more homogenous hardness values as indicated by Figure 2(b). The relevant data map before and after filtration process is shown in Figure 3.

Many statistical features are applied to statistically examine the data nature and significance. As listed in Table 3, standard deviation is improved for most sectors as well as for the entire set of data. Improvement was reflected in the values of standard deviation parameters of all layers, Table 4, where a reduction of about 74, 43 and 58 percent is attained for layers numbers 1, 2 and 3 respectively. As seen by *T*-test analysis, Table 5, filtered data indicated not only lower standard deviation value but also more compact 95% confidence interval and standard error of estimates with higher *t*-value. It is observed that the data was principally affected by the impact of the low measured hardness values. Table 6 summarizes the statistical *T*-test Pairs, One-way ANOVA and Correlation Statistical parameters for filtered and unfiltered data. T-test pairs analysis indicates that the layer sequence may exhibit a possible slight correlation in such a way that lower specimen diameter seems to be harder.

**Table 3:** MEANS statistical parameters of Hi-sector interaction.

Sector	Filtered data			Unfiltered data		
	Mean	N	Std. Dev.	Mean	N	Std. Dev.
1	79.20	1	.	76.20	3	3.89
2	81.60	2	3.68	84.03	3	4.95
3	-	-	-	85.97	3	12.78
4	82.63	3	2.74	82.63	3	2.74
5	82.23	3	5.25	82.23	3	5.25
6	85.15	2	1.20	79.40	3	9.995
7	84.73	3	2.95	84.73	3	2.95
8	82.45	2	1.20	78.70	3	6.55
Total	82.90	16	3.09	81.74	24	6.64

**Table 4:** Means statistical parameters of Hi-layer interaction.

Layer	Filtered data			Unfiltered data		
	Mean	N	Std. Dev.	Mean	N	Std. Dev.
1	84.50	5	2.01	81.55	8	7.71
2	83.47	6	3.12	82.66	8	5.53
3	80.62	5	3.07	81.00	8	7.31
Total	82.90	16	3.09	81.74	24	6.64

**Table 5:** *T*-test statistical parameters for filtered and unfiltered data.

N	Mean	Std. Dev.	Std. Error	t-value	Sig.	95% Conference interval	
						Lower	upper
16	82.9	3.09	0.7726	107.3	0.00	81.25	84.55
24	81.74	6.64	1.356	50.27	0.00	78.93	84.54

**Table 6:** *T*-test pairs, one-way ANOVA and correlation statistical parameters.

Layer	Filtered data					Unfiltered data				
	N	Corr.	Sig.	One-way ANOVA		N	Corr.	Sig.	One-way ANOVA	
				F-ratio	Sig.				F-ratio	Sig.
Pair 1 (Hi & Sector)	16	0.364	0.165	0.566	0.749	24	0.004	0.986	0.621	0.691
Pair 2 (Hi & Layer)	16	−0.513	0.042	2.581	0.114	24	−0.035	0.873	0.121	0.887

### 3.2. Hardness Variability of AISI 4140 Steel

**Figure 4:**
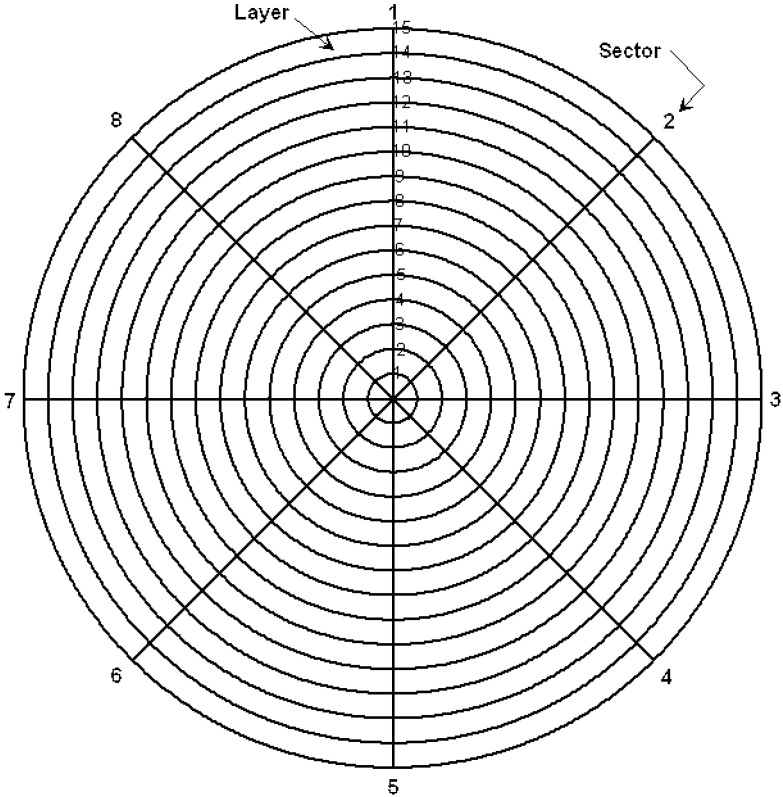
Stratifying design for hardness measurements over cross section surface.

Based on the evolved outcome and findings, a stratified design over the cross-section circular faces of AISI 4140 is proposed as shown in Figure 4. Eight circumferential sectors of 45 deg angular space and fifteen radial layers have led to a total of 120 data readings located at the circumference-radial lines intersections as shown in Figure 4.

Statistical features of the measured hardness values over both cross-section sides A and B along with their aggregated set are listed in Table 7. Although a significant statistical judgment is observed for readings taken on side B, the use of the entire data for both sides seems to improve the significance and the adequacy of the data set.

**Table 7:** Statistical analysis for entire data of both sides of AISI 4140 (H_m_ = HRB 99).

Side	Range		Mean	Std. Dev.	Std. Error	t-value	Sig.	95% Conference interval	
	Min	Max						Lower	Upper
A	89	110.7	100.8	6.79	0.619	162.8	0.000	99.57	102.03
B	90	111.3	105.9	4.61	0.421	251.7	0.000	105.07	106.74
A+B	89	111.3	103.35	6.33	0.408	253.0	0.000	102.55	104.16

When data for each side is individually dealt with, the “Detect Anomaly” analysis, with the specified criterion measures, as in the last section, suggested the exclusion removal of six data points from each side. However, when data for both sides were considered, ten cases from side B and two cases from side A were suggested for exclusion. As indicated in Table 8, statistical measures are rarely affected after outlier removal. With increasing data points, it is expected that the outlier impact is diluted and this justifies the emerged trend for the reference block when only 24-cases are considered.

**Figure 5:**
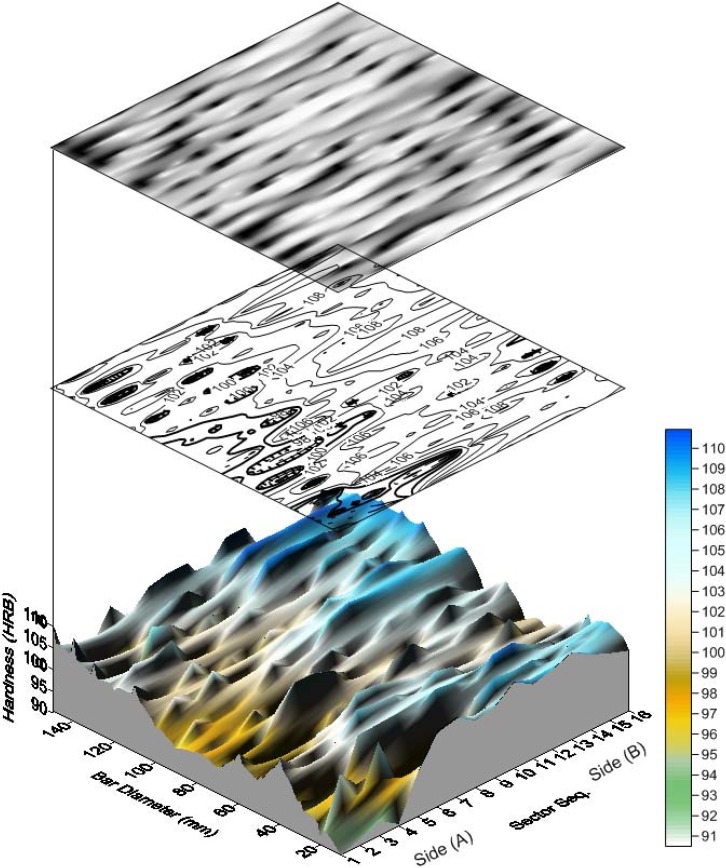
Surface map, contour and response surface of both sides.

**Figure 6:**
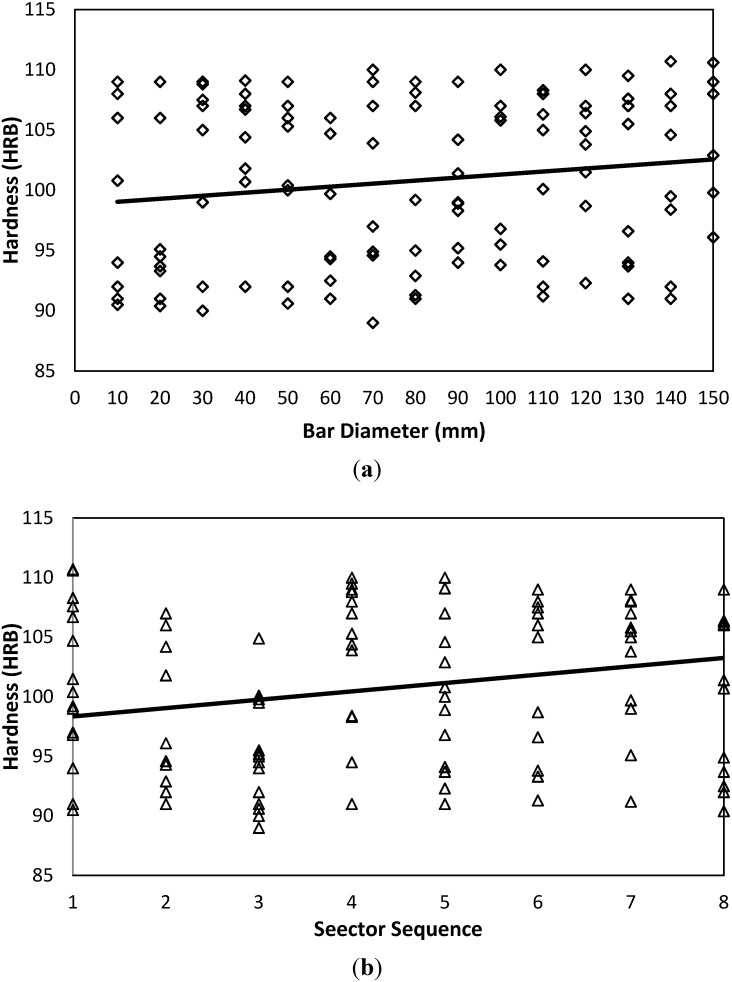
Hardness-Diameter-Sector sequence interrelations for side A. (**a**) Hardness-Diameter-AISI 4140-Side A; (**b**) Hardness-Sector-AISI 4140-Side A.

**Figure 7:**
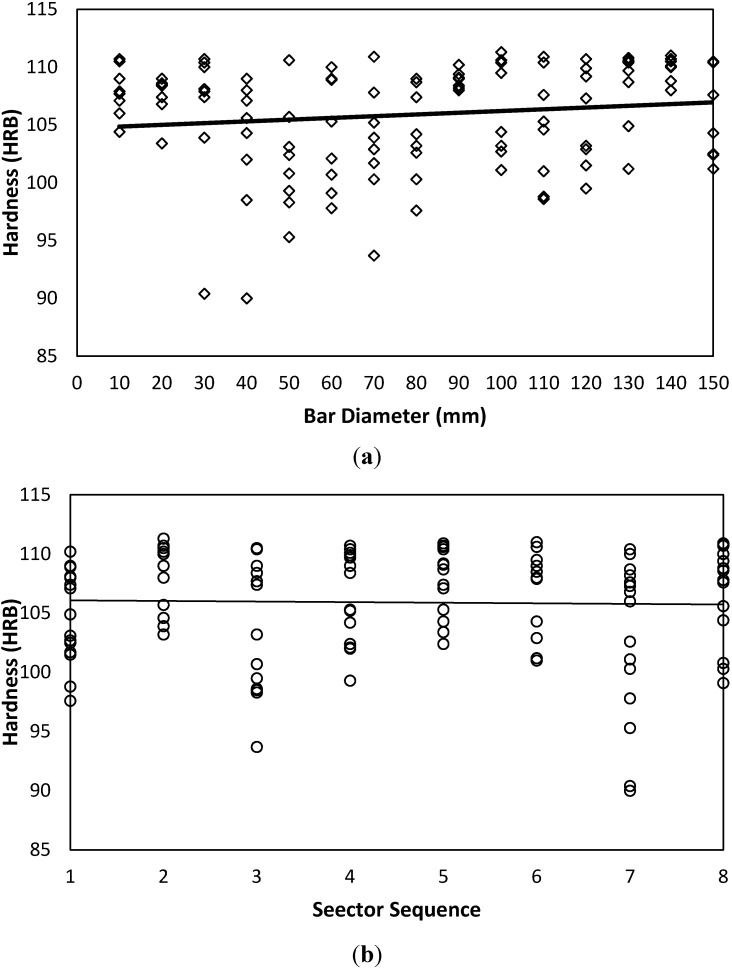
Hardness-Diameter-Sector sequence interrelations for side B. (**a**) Hardness-Diameter-AISI 4140-Side B; (**b**) Hardness-Sector-AISI 4140-Side B.

Figure 5 shows surface map, surface contour and response surface of the entire data of both section sides. This is accompanied by a color scale to indicate the hardness levels as judged by the color intensity. A hardness increasing trend at larger diameters or outer layers is observed over both sides. Also, it is generally shown that over side B, the recorded hardness values have greater values (compare mean values of both sides in Table 7 and Table 8). Possible trends are shown when the plain raw data are plotted as in Figure 6 and Figure 7. A slight positive trend and dependency on the bar diameter is detected; see Figure 6(a) and Figure 7(a). However, as far as the hardness-sector sequence is concerned, (Figure 6(b) and Figure 7(b)), there is no solid evidence against the assumable random nature of the measured data. All the aforementioned findings are reflected by the statistical criteria as listed in Table 9 using T-test Pairs, One-Way ANOVA and the partial correlation criteria.

**Table 8:** Statistical analysis after outlier inclusion of AISI 4140 (H_m_ = HRB 99).

Side	Range		Mean	Std. Dev.	Std. Error	t-value	Sig.	95% Conference interval	
	Min	Max						Lower	Upper
A	89	110.6	100.7	6.90	0.646	155.9	0.000	99.40	101.97
B	95.3	111.3	106.3	4.07	0.381	279.0	0.000	105.50	107.01
A+B	89	111.3	103.4	6.49	0.430	240.0	0.000	102.56	104.25

**Table 9:** *T*-test pairs, one-way ANOVA and correlation statistical parameters.

Layer	Side A					Side B				
	N	Correlation	Sig.	One-way ANOVA		N	Correlation	Significance	One-way ANOVA	
				F-ratio	Sig.				F-ratio	Sig.
Pair 1 (Hi & Diameter)	120	0.160	0.080	0.983	0.476	120	0.141	0.125	2.497	0.004
Pair 2 (Hi & Sector #)	120	0.238	0.009	4.502	0.000	120	−0.025	0.789	2.359	0.028

### 3.3. Hardness Variability of AISI 1020 Steel and AA 6082 Aluminum Alloy

To attain more universal conclusions and to verify the aforementioned findings, the same experimental procedures were carried out considering different materials with different hardness levels, chemical composition and mechanical properties, as indicated in Table 1.

**Figure 8:**
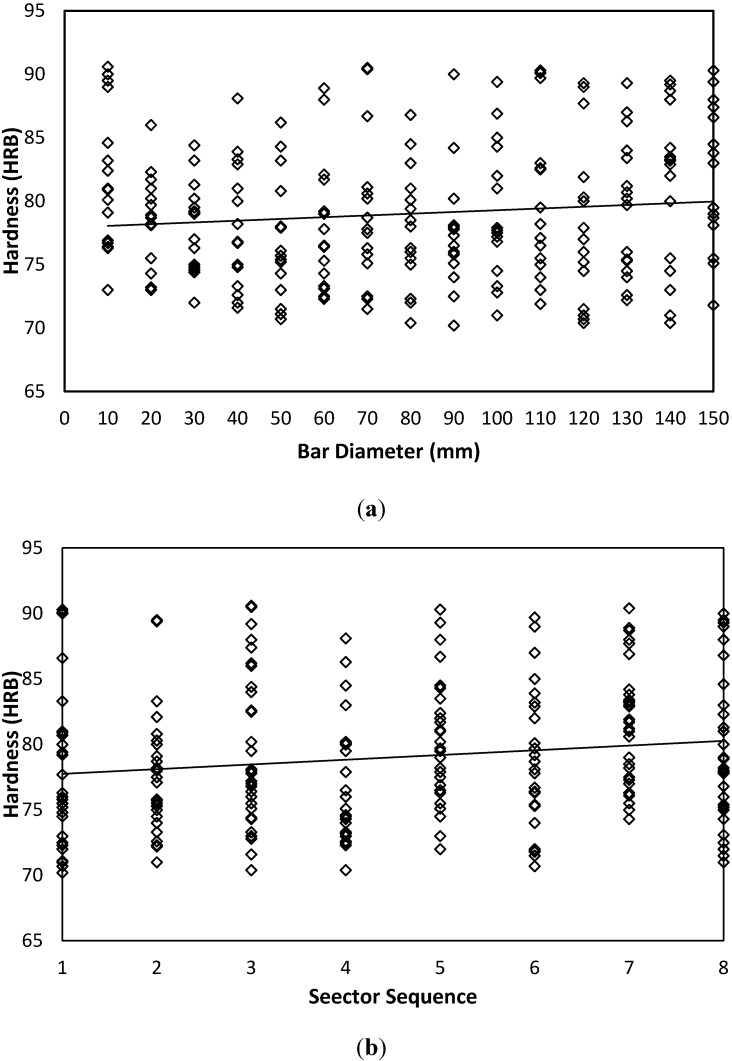
Hardness variability as affected by bar diameter and sector sequence of AISI 1020. (**a**) Hardness-Diameter-AISI 1020-Both Sides; (**b**) Hardness-Sector-AISI 1020-Both Sides.

**Figure 9:**
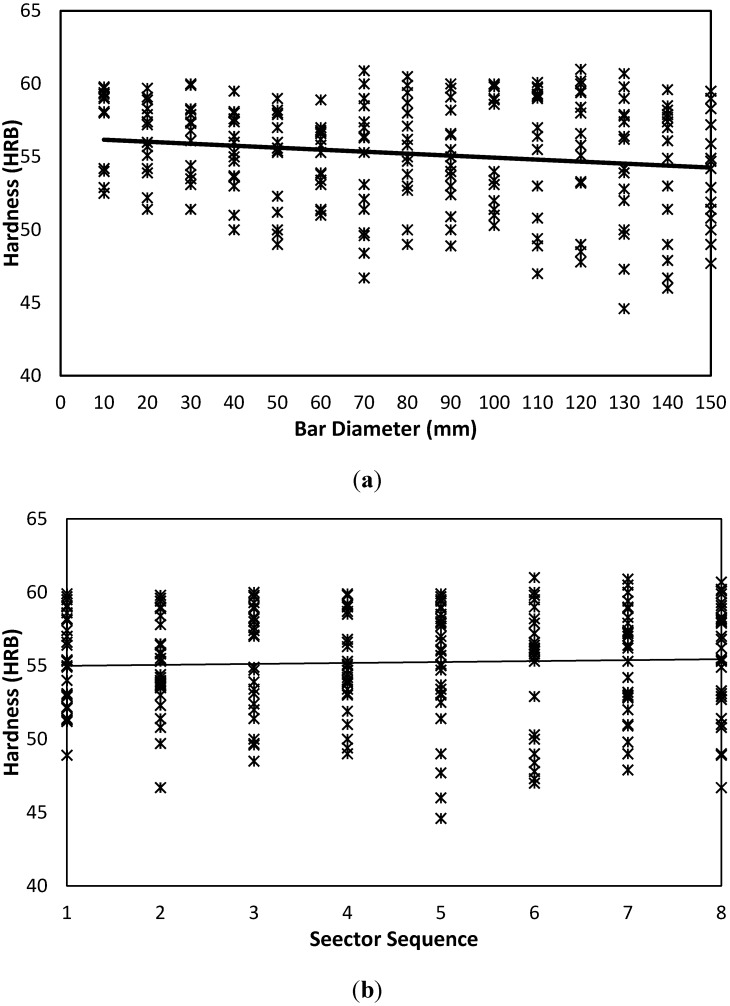
Hardness variability as affected by bar diameter and sector sequence of AA 6082 Aluminum Alloy. (**a**) Hardness-Diameter-AA 6082-Both Sides. (**b**) Hardness-Sector-AA 6082-Both Sides.

Hardness measured values for AISI 1020 carbon steel are shown in Figure 8. Data distribution over both sides indicates a stochastic nature with tight confidence interval and mean. For AISI 1020, a similar qualitative trend to that of AISI is observed, where it is shown that hardness tends to have a slight and insignificant increasing trend with each bar diameter; see Figure 8(a), and sector sequence, Figure 8(b). However, for AA 6082 aluminum alloy, Figure 9 data shows a slight negative trend with the bar diameter parameter, Figure 9(a).

## 4. Conclusions

Hardness homogeneity represents a key factor for a robust design considering the durability and reliability of most manufactured engineering products. In this context, the object was to examine the hardness variability of some ferrous and nonferrous structural engineering materials; AISI 1020 and AISI 4140 quenched and tempered hot alloy steel, as well as AA6082 aluminum alloy. Additionally, the measuring credibility of the reference block was examined.

Measurements were performed according to a stratified design covering the section’s entire area both in diagonal and circumferential directions. Hardness values were analyzed using appropriate statistical and graphical measures.

Generally, a wide variability was observed in the measured hardness values using the current press-and-read digital portable tester, compared to those that may be obtained using conventional stationary instruments using 100 kg (9800 N) load and 1/16″ steel ball. Lower variability levels were obtained when the calibration was carried out using the provided reference block. Higher variability levels for test pieces may be mainly due to the material inhomogeneity along with the sample size used throughout the analysis (bigger data points statistically leads to wider scatter). Statistical parameters for the data over the apparatus reference block proved the reliability of the measuring system, where no strong evidence was found against the stochastic nature of hardness measures over the various stratified locations. Also, outlier elimination procedures were proved to be beneficial when a few, but still a sufficient number, of the measured points are considered.

For all the materials tested with sufficient data, either from single or both section sides, a stochastic hardness pattern was observed with a dispersion domain that is within the acceptable confidence interval. However, for AISI 4140 and AISI 1020, a slight correlation trend was observed indicating lower hardness values toward the bars section center. A contradictory trend was detected for AA 6082 aluminum alloy. However, no definite significant behavior was noticed regarding the effect of the sector sequence.
